# Relationship between lipid metabolism, coagulation and other blood indices and etiology and staging of non-traumatic femoral head necrosis: a multivariate logistic regression-based analysis

**DOI:** 10.1186/s13018-024-04715-x

**Published:** 2024-04-20

**Authors:** Ximing Yu, Shilu Dou, Liaodong Lu, Meng Wang, Zhongfeng Li, Dongwei Wang

**Affiliations:** 1 The First Clinical College of Shandong University of Traditional Chinese Medicine, Jinan, 252000 China; 2 Department of Orthopedics, Liaocheng City Hospital of Traditional Chinese Medicine, Liaocheng, 252000 China

**Keywords:** Non-traumatic necrosis of the femoral head, Risk factor, Coagulation indicators, Biochemical indicators

## Abstract

**Background:**

To analyze the relationship between lipid metabolism, coagulation function, and bone metabolism and the contributing factor and staging of non-traumatic femoral head necrosis, and to further investigate the factors influencing the blood indicators related to the staging of non-traumatic femoral head necrosis.

**Methods:**

The medical records of patients with femoral head necrosis were retrieved from the inpatient medical record management system, and the lipid metabolism, bone metabolism, and coagulation indices of non-traumatic femoral head necrosis (including alcoholic, hormonal, and idiopathic group) were obtained according to the inclusion and exclusion criteria, including Low-Density Lipoprotein Cholesterol, Triglycerides, Non-High-Density Lipoprotein Cholesterol, Apolipoprotein A1, Apolipoprotein (B), Apolipoprotein (E), Uric Acid, Alkaline Phosphatase, Bone-specific Alkaline Phosphatase, Activated Partial Thromboplastin Time, Prothrombin Time, D-dimer, Platelet count. The relationship between these blood indices and the different stages under different causative factors was compared, and the factors influencing the stages of non-traumatic femoral head necrosis were analyzed using multivariate logistic regression.

**Results:**

(i) Gender, Age and BMI stratification, Low-density Lipoprotein Cholesterol, Triglycerides, Non-High-density Lipoprotein Cholesterol, Apolipoprotein (B), Apolipoprotein (E), Uric Acid, Bone-specific Alkaline Phosphatase, Activated Partial Thromboplastin Time, Plasminogen Time, D-dimer, and Platelet count of the alcohol group were statistically different when compared among the different ARCO staging groups; (ii) The differences in Age and BMI stratification, Triglycerides, Non-High-density Lipoprotein Cholesterol, Apolipoprotein A1, Apolipoprotein B, Apolipoprotein E, Uric Acid, Bone-specific Alkaline Phosphatase, Activated Partial Thromboplastin Time, Plasminogen Time, D-dimer, and Platelet count were statistically significant when compared among the different phases in the hormone group (*P* < 0.05); (iii) The differences in Age and BMI stratification, Non-High-Density Lipoprotein Cholesterol, Apolipoprotein A1, Apolipoprotein (B), Apolipoprotein (E), Uric Acid, Activated Partial Thromboplastin Time, D-dimer, and Platelet count were statistically significant when compared among the different stages in the idiopathic group (*P* < 0.05); (v) Statistically significant indicators were included in the multivariate logistic regression analysis, excluding the highly correlated bone-specific alkaline phosphatase, and the results showed that Low-density lipoprotein was negatively correlated with changes in the course of ARCO, and Non-High-Density Lipoprotein cholesterol, Apo B, Activated Partial Thromboplastin Time, and Platelet count were significantly and positively correlated with disease progression.

**Conclusion:**

An abnormal hypercoagulable state as well as an abnormal hyperlipidemic state are risk factors for the progression of non-traumatic femoral head necrosis under various exposure factors, as indicated by Non-High-Density Lipoprotein Cholesterol, Apolipoprotein B, Activated Fractional Thromboplastin Time, and Platelet Counts.

**Supplementary Information:**

The online version contains supplementary material available at 10.1186/s13018-024-04715-x.

## Introduction

Osteonecrosis of the femoral head (ONFH) is a common clinical disease with a complex etiology, in which insufficient blood supply to the femoral head is the key causative factor for its development [1]. When it is found in the clinic, it is usually characterized by structural changes of the femoral head and different degrees of collapse, accompanied by pain and activity limitation of the affected side of the hip joint, which ultimately leads to the loss of hip function and seriously affects the quality of life [2]. Non-traumatic necrosis of the femoral head (NONFH) has a high incidence in middle and young-aged people, and a study in the late 1990s showed that 10,000–20,000 new patients were diagnosed with NONFH in the United States each year [3], and according to a recent epidemiologic statistic [4], the number of cases of NONFH in the Chinese population aged 15 years and older is forecasted to be 8.12 million. Therefore, clarifying the pathogenesis and targeting interventional therapy to slow down the progression of the disease is a key concern for clinicians. Currently, the etiology of NONFH has been considered to include coagulation disorders, abnormal lipid metabolism, oxidative stress, fat embolism, vascular endothelial disorders as well as thrombosis, diabetes mellitus, and genetic variation [5, 6], and the main risk factors and estimated frequencies include Steroid-induced necrosis of the femoral head (SONFH) with long-term use of steroids (35–40%), alcoholic osteonecrosis of the femoral head(AONFH) with chronic heavy alcohol use (20–40%) and idiopathic osteonecrosis of the femoral head(IONFH)(20–40%) [7]. In addition, due to the rapid progression of NONFH, the clinical diagnosis is mainly made by patients’ symptomatic complaints and imaging manifestations, and there is no accurate method or index for the clinical detection of each stage of NONFH. Studies have shown that some blood indicators can be used as clinical indicators to monitor the progression of NONFH [8], and the model is stable by constructing a prediction model to analyze indicators such as total cholesterol level, triglyceride level, white blood cell count, gender and platelet count, which is used for early screening and diagnosis of NONFH. Currently, Some studies have investigated the correlation between lipid metabolism and coagulation abnormalities and the development of femoral head necrosis, but there are no reports on the relationship between lipid metabolism, coagulation function, and bone metabolism-related indexes and the etiology of NONFH and its staging, so it is necessary to further investigate this situation, to clarify the impact of lipid metabolism, bone metabolism and coagulation disorders on the process of NONFH. The study is necessary to further investigate this situation to clarify the effects of lipid metabolism, bone metabolism, and coagulation disorders on the progression of NONFH.

## Methods

### Participants

This study investigated the medical records of 1052 NONFH patients admitted to the Department of Bone and Joint of Liaocheng City Hospital of Traditional Chinese Medicine affiliated Shandong University of Traditional Chinese Medicine(SDUTCM) from June 2019 to June 2023, and the case inclusion criteria were referred to the diagnostic criteria of NONFH in the Guidelines for Integrative Diagnosis and Treatment of NONFH in Western and Eastern Medicine issued by the Chinese Society of Traditional Chinese Medicine in 2023 [9], (1) the clinical features were characterized by predominantly pain in the hip, buttock or groin area, occasionally accompanied by knee pain and limited hip internal rotation; (2) diagnosis of NONFH by X-ray, MRI and CT manifestations; and (3) absence of direct trauma, cardiovascular and cerebrovascular diseases, ankylosing spondylitis, metabolic disorders or bone metastases. In addition to this according to ZHAO [4]’s description of the pathogenesis of NONFH, according to the history of alcohol overdrinking(alcohol overdrinking [10] were defined as drinking ≥ 40 g of alcohol per day for men and ≥ 20 g for women in the past 12 months), hormone overdos(hormone overdos [11] was defined as ≥ 2 g per day of systemic steroids including prednisone or other isotonic drugs in the past 3 months), and no specific history of the disease were splited into the alcohol group, hormone group, and idiopathic group. The exclusion criteria were (1) incomplete medical records or not the first diagnosis of NONFH; (2) incomplete clinical data; (3) involved serious diseases of vital organs or accompanied by malignant tumors; (4) previous medication for severe hepatic impairment or steatohepatitis due to heavy alcohol consumption and hormone overdose. 260 cases in the alcohol group, 283 cases in the hormone group, and 252 cases in the idiopathic group were obtained, and the combination of Association Research Circulation Osseous (ARCO) 2019 ONFH Combined with the ARCO 2019 ONFH staging criteria [12], we finally obtained 44 cases of ARCO stage II, 111 cases of stage III, and 105 cases of stage IV in the alcohol group; 63 cases of ARCO stage II, 132 cases of stage III, and 88 cases of stage IV in the hormone group; and 42 cases of ARCO stage II, 119 cases of stage III, and 91 cases of stage IV in the idiopathic group.

### Clinical data

This retrospective, single-center observational study extracted general information from the inpatient medical record management system for all patients on admission, including age, sex, region, BMI [kg/m2], and history of smoking and alcohol consumption; and extracted all blood laboratory markers after fasting for at least 10 h, including Low-density Lipoprotein Cholesterol (LDL), Triglycerides (TG), Non-High-density Lipoprotein Cholesterol (serum Total Cholesterol-High-density Lipoprotein cholesterol) (Non-HDL), Apolipoprotein A1 (ApoA1), Apolipoprotein B (ApoB), Apolipoprotein E (ApoE), Uric Acid (UA), Alkaline Phosphatase (ALP), Bone-specific Alkaline Phosphatase (b-ALP), Activated Artial Thromboplastin Time (APTT), Prothrombin Time (PT), D-dimer (D-D), and Platelet count (PLT). Blood laboratory parameters of patients with different ARCO stages in each etiological group were analyzed, and further logistic regression analysis was performed to analyze the independent risk factors for the course of NONFN.

### Ethical aspects

This study strictly adhered to the Declaration of Helsinki and was approved by the Ethics Committee of Liaocheng City Hospital of Traditional Chinese Medicine (No. LLW2024003). All patients gave informed consent to participate in this study, and all experimental data were used only by the investigators for disease research, and any public reporting of the results of this study will not reveal any private information.

### Statistical analysis

IBM SPSS Statistics version 26.0 was used for statistical analysis. Shapiro-Wilk was used to test the normality of the data.Kruskal-Wallis H test was used to compare continuous data in multiple groups.Two-way comparisons were analyzed and evaluated using the Mann-Whitney U test. Categorical variables were analyzed using the chi-squared test.All statistical analyses were performed using two-tailed tests, and differences were considered significant at *P* < 0.05.Normally distributed measures are expressed as mean ± SD, and non-normally distributed measures are expressed as median and quartiles [M(Q1, Q3)].All statistically significant indicators were included in Spearman’s correlation analysis, and after excluding highly correlated laboratory indicators, the remaining results were subjected to multivariate logistic regression to analyze the correlates of NONFH, and the level of the test was set at α = 0.05, which ultimately yielded the risk factors for the course of NONFH.

## Results

### Demographic characteristics of the study participants

A total of 795 cases of NONFH patients were included, including 260 cases in the alcohol group, 283 cases in the hormone group and 252 cases in the idiopathic group, and the following results were obtained by analyzing the gender, age and BMI of different ARCO stages among the groups: there were more male patients than female patients in different stages in the alcohol group, and the difference was statistically significant (*P* < 0.05), With regard to age, the majority of people aged 45–59 years in ARCO stages III and IV and the majority of people under 45 years in stage II, the difference was significant (*P* < 0.001), ARCO stage II and III with BMI < 25 kg/m2 majority, ARCO stage IV BMI ≥ 25 kg/m2 accounted for 58.1%, the difference was significant (*P* < 0.001); hormone group and the idiopathic group of the different ARCO staging between the sexes compared were not statistically significant (*P* = 0.05); hormone group and the idiopathic group of the different ARCO staging of gender comparison of the differences are not statistically In the hormone group, the age groups with different stages were 60 years old and above (47.6%), 45 years old and above (38.6%), and 45–59 years old and above (81.8%), while the age groups with different stages in the idiopathic group were 45 years old and above (50%), 60 years old and above (38.4%), and 60 years old and above (38.4%), and in the idiopathic group were 60 years old and above (38.4%). (38.4%), stage IV 45–59 years old (85.7%), the difference between the two groups is significant (*P* < 0.001); BMI hormone group III, IV stage BMI 25 kg/m2 or more accounted for more (*P* < 0.001), the special group II, III stage BMI25kg/m2 or less accounted for more (*P* < 0.001). (Table 1)

**Table 1:** Comparison of the basic conditions of patients in the three groups

Variable		ARCO II	ARCO III	ARCO IV	*P* value
Alcohol group	Sex				0.001
	Male	27(61.4%)	91(82.0%)	92(87.6%)	
	Female	17(38.6%)	20(18.0%)	13(12.4%)	
	Age				<0.001
	<45	27(61.4%)	32(28.8%)	19(18.1%)	
	45–59	4(9.1%)	45(40.5%)	74(70.5%)	
	≥ 60	13(29.5%)	34(30.6%)	12(11.4%)	
	BMI kg/m^2^				<0.001
	<25	39(88.6%)	76(68.5%)	44(41.9%)	
	≥ 25	5(11.4%)	35(31.5%)	61(58.1%)	
Hormone group	Sex				0.357
	Male	19(30.2%)	53(40.2%)	30(34.1%)	
	Female	44(69.8%)	79(59.8%)	58(65.9%)	
	Age				<0.001
	<45	28(44.4%)	51(38.6%)	8(9.1%)	
	45–59	5(7.9%)	47(35.6%)	72(81.8%)	
	≥ 60	30(47.6%)	34(25.8%)	8(9.1%)	
	BMI kg/m^2^				<0.001
	<25	58(92.1%)	66(50.0%)	33(37.5%)	
	≥ 25	5(7.9%)	66(50.0%)	55(62.5%)	
Idiopathic group	Sex				0.810
	Male	22(52.4%)	66(55.5%)	53(58.2%)	
	Female	20(47.6%)	53(44.5%)	38(41.8%)	
	Age				<0.001
	<45	21(50.0%)	34(28.6%)	5(5.5%)	
	45–59	2(4.8%)	39(32.8%)	78(85.7%)	
	≥ 60	19(45.2%)	46(38.7%)	8(8.8%)	
	BMI kg/m^2^				<0.001
	<25	40(95.2%)	81(68.1%)	41(45.1%)	
	≥ 25	2(4.8%)	38(31.9%)	50(54.9%)	

Date are presented as n(%)

### Grouped single factor analysis of laboratory indicators

Only TG in the alcohol group conformed to a normal distribution for the whole group, the rest were continuous variables and did not conform to the normal distribution, analyzed by Kurskai-Wallis H-test, and LDL, N-HDL, TG, ApoB, UA, ApoE, b-ALP, APTT, PT, D-D, and PLT factors were compared between different ARCO staging groups The difference was statistically significant (Table 2). LDL and PLT in the hormone group conformed to normal distribution, the rest did not, and the differences between N-HDL, TG, ApoB, ApoA1, UA, ApoE, b-ALP, APTT, PT, D-D, and PLT were tested to be statistically significant when compared among different staging groups (*P* < 0.01) (Table 3). Only PLT in the idiopathic group conformed to normal distribution, and the analysis revealed statistically significant differences in N-HDL, ApoB, ApoA1, UA, ApoE, APTT, D-D, and PLT when comparing them between different ARCO staging periods (Table 4).

**Table 2:** Comparison of laboratory indices in patients with different ARCO stages in the alcohol group

Variable	ARCO II	ARCO III	ARCO IV	Test value	*P* value
LDL mmol/L	3.14(2.68,3.65)	3.38(2.82,3.89)^b^	3.42(2.91,3.96)	6.24	0.044
N-HDL mmol/L	3.43(3.01,3.98)	4.08(3.56,4.62)	4.41(3.87,4.94)	56.42	<0.001
TG mmol/L	1.57 ± 0.67	1.67 ± 0.56^b^	1.90 ± 0.58	9.69	0.008
ApoB g/ml	1.02(0.89,1.24)	1.23(0.99,1.44)	1.32(1.15,1.59)	41.57	<0.001
ApoA1 g/ml	1.31(1.12,1.64)	1.29(1.17,1.37)	1.24(1.02,1.38)	4.95	0.084
UA umol/L	367.75(347.00,397.55)	422.30(394.00,452.50)	469.00(409.00,527.90)	38.89	<0.001
ApoE mg/dl	3.47(2.93,4.46)	4.23(3.34,5.44)	4.95(3.60,6.12)	24.75	<0.001
ALP U/L	65.10(50.75,83.25)	71.00(54.82,87.53)	71.50(58.00,86.01)	3.57	0.168
b-ALP U/L	38.50(25.00,53.00)	45.00(31.00,59.00)	38.00(25.00,51.00)^a^	14.71	0.001
APTT s	23.43(21.98,24.30)	25.56(24.37,36.57)	23.90(22.48,25.35)	41.27	<0.001
PT s	10.51(9.65,11.34)	11.13(9.94,12.33)	10.10(9.13,11.09)^a^	28.14	<0.001
D-D ug/ml	0.33(0.26,0.37)	0.51(0.43,0.63)	1.14(0.98,1.30)	184.44	<0.001
PLT 10^9^/L	198.14 ± 23.80	224.12 ± 41.97	187.02 ± 32.95^a^	45.17	<0.001

Date are presented as M[Q1,Q3] or mean ± SD;^a^*P* >0.05 compared with ARCO stage II; ^b^*P* >0.05 compared with ARCO stage II

**Table 3:** Comparison of laboratory indices in patients with different ARCO stages in the hormone group

Variable	ARCO II	ARCO III	ARCO IV	Test value	*P* value
LDL mmol/L	3.12 ± 0.47	3.27 ± 0.57	3.31 ± 0.58	4.41	0.11
N-HDL mmol/L	3.24(2.94,3.58)	3.81(3.41,4.40)	4.26(3.78,4.73)	83.90	<0.001
TG mmol/L	1.10(0.83,1.47)	1.46(0.89,1.98)	1.71(1.14,2.32)	24.92	<0.001
ApoB g/ml	1.03(0.92,1.24)	1.18(0.96,1.4)	1.3(1.02,1.49)	25.02	<0.001
ApoA1 g/ml	1.1(0.94,1.26)	1.29(1.00,1.47)	1.35(1.09,1.49)	23.77	<0.001
UA umol/L	318.00(290.80,347.50)	356.85(331.45,383.90)	384.75(360.20,407.15)	51.11	<0.001
ApoE mg/dl	3.34(3.03,4.05)	4.01(3.09,5.64)	4.70(3.29,6.92)	24.77	<0.001
ALP U/L	69.00(53.33,84.67)	72.25(55.26,88.75)	72.25(60.71,85.31)	2.58	0.276
b-ALP U/L	38.00(25.00,52.00)	42.50(32.50,53.50)	37.00(23.50,51.00)^a^	9.30	0.01
APTT s	24.28(23.10,25.45)	26.22(25.10,27.57)	26.09(24.49,27.17)	29.58	<0.001
PT s	10.80(9.91,11.70)	11.6(10.71,12.5)	10.45(9.38,11.43)^a^	51.73	<0.001
D-D ug/ml	0.32(0.23,0.38)	0.53(0.43,0.65)	1.04(0.92,1.13)	197.31	<0.001
PLT 10^9^/L	213.65 ± 31.41	236.33 ± 46.31	197.98 ± 36.53	36.17	<0.001

Date are presented as M[Q1,Q3] or mean ± SD;^a^*P*>0.05compared with ARCO stage II;^b^*P* >0.05 compared with ARCO stage II

**Table 4:** Comparison of laboratory indices in patients with different ARCO stages in the idiopathic group

Variable	ARCO II	ARCO III	ARCO IV	Test value	*P* value
LDL mmol/L	2.98(2.30,3.63)	3.07(2.60,3.57)	3.14(2.58,3.72)	1.88	0.391
N-HDL mmol/L	3.33(2.52,3.93)	3.66(3.20,4.10)	3.96(3.45,4.53)	30.02	<0.001
TG mmol/L	1.21(0.71,1.75)	1.34(0.96,1.77)	1.41(0.71,2.04)	1.90	0.987
ApoB g/ml	0.99(0.90,1.15)	1.13(0.99,1.29)	1.18(1.00,1.46)	24.06	<0.001
ApoA1 g/ml	1.20(0.98,1.36)	1.24(1.00,1.41)^b^	1.38(1.22,1.52)	30.32	<0.001
UA umol/L	326.45(288.60,352.60)	342.00(300.70,383.00)^b^	357.60(311.90,404.60)	9.64	0.008
ApoE mg/dl	3.11(2.48,4.11)	3.59(3.03,4.38)	4.31(3.64,6.73)	37.46	<0.001
ALP U/L	69.71(53.95,84.91)	71.00(56.30,85.70)	72.00(55.90,88.10)	0.74	0.692
b-ALP U/L	41.00(26.50,56.50)	43.00(28.00,57.00)	41.00(28.00,54.00)	1.21	0.545
APTT s	25.10(24.11,26.27)	26.04(24.98,27.25)	26.23(24.96,28.03)	12.42	0.002
PT s	10.77(9.65,11.91)	11.05(9.68,12.31)	10.70(9.65,11.75)	4.20	0.123
D-D ug/ml	0.26(0.19,0.35)	0.42(0.33,0.53)	0.75(0.60,0.91)	146.29	<0.001
PLT 10^9^/L	204.9 ± 31.03	227.25 ± 39.22	200.1 ± 41.08^a^	25.11	<0.001

Date are presented as M[Q1,Q3] or mean ± SD;^a^*P* >0.05 compared with ARCO stage II; ^b^*P* >0.05 compared with ARCO stage II

### Multivariate Logistic Regression Analysis

ARCO stage II was used as a control, and age, gender, and BMI were assigned to illustrate age, etiology, body mass, and gender: age (1 = < 45 years, 2 = 45–59 years, 3 = ≥ 60 years), etiology (1 = alcoholic, 2 = hormonal, and 3 = idiopathic), gender (male = 1, female = 0), and defining a BMI of ≥ 25 kg/m2 as overweight (yes = 0, no = 1 ), Spearman’s correlation analysis was performed for the indicators that were statistically different (*P* < 0.05) in the univariate analysis. b-ALP showed a high correlation with other indicators at the 0.05 level, so b-ALP was excluded, and LDL, N-HDL, TG, ApoB, ApoA1, UA, ApoE, and APTT were included. pt, D-D, and PLT were analyzed by multivariate Logistic regression analysis. The results suggested that the model chi-square value was 1433.064, with a significance of < 0.001; the three pseudo-R2 values of Cox and Snell, Nagelkerke, and Mc Fadden were 0.836, 0.955, and 0.868, which indicated a good model fit, and the model had the highest accuracy in predicting the stage III course of ARCO, which amounted to 98.1%, and the overall prediction accuracy was 97.4%.

Comparing ARCO stage III with stage II, LDL, TG, and ApoA1 were negatively correlated with ARCO stage III onset, suggesting that the risk of ARCO stage III onset was lower than that of stage II against the background of these indexes (*P* < 0.05), with the corresponding OR and 95% CL of 0.153 (95% CL: 0.09–0.259, *P* < 0.001), 0.87 (95% CL. 0.611–1.239,*P* = 0.44), 0.654 (95% CL: 0.481–0.889, *P* = 0.007); N-HDL, ApoB, UA, APTT, PT, PLT, and age 45–59 years were positively correlated with the onset of Stage III, with an increased probability of onset, corresponding to an OR and 95% CL of 5.000 (95% CL. 3.445–7.257, *P* < 0.001), 5.009 (95% CL: 2.572–9.754, *P* < 0.001), 1.008 (95% CL: 1.000-1.016, *P* = 0.048), 1.336 (95% CL: 1.063–1.679, *P* = 0.013), 1.738 (95% CL: 1.143–2.642, *P* = 0.01), 1.024 (95% CL: 1.009–1.039, *P* = 0.002), and 50.406 (95% CL: 8.941-284.172, *P* < 0.001).ARCO stage IV vs. stage II, LDL and BMI < 25 kg/m2 suggested an association with reduced risk of morbidity (*P* < 0.05), N-HDL, ApoB, APTT, D-D, and age 45–59 years wererelated to the increased risk of morbidity, and their OR and 95% CL were 16.592 (95% CL:10.886–25.287,*P* < 0.001), 16.63 (95% CL:7.731–35.776,*P* < 0.001) respectively, 1.335 (95% CL: 1.061–1.678, *P* = 0.013), 3.227 (95% CL: 2.221–4.668, *P* < 0.001), 571.533 (95% CL: 84.785-3852.674, *P* < 0.001). (Table 5)

**Table 5:** ARCO Multivariate Logistic Regression Analysis for Different Stages of ARCO

		B	Wald	*P* value	Exp(B)	95%CL	
						Lower	Upper
III	Intercept	−29.47	19.485	<0.001			
	LDL	−1.88	48.193	<0.001	0.153	0.09	0.259
	N-HDL	1.609	71.742	<0.001	5	3.445	7.257
	TG	−0.139	0.596	0.44	0.87	0.611	1.239
	ApoB	1.611	22.454	<0.001	5.009	2.572	9.754
	ApoA1	−0.425	7.346	0.007	0.654	0.481	0.889
	UA	0.008	3.927	0.048	1.008	1	1.016
	ApoE	0.204	1.803	0.179	1.226	0.91	1.652
	APTT	0.289	6.157	0.013	1.336	1.063	1.679
	PT	0.553	6.678	0.01	1.738	1.143	2.642
	D-D	0.288	2.913	0.088	1.333	0.958	1.855
	PLT	0.023	9.667	0.002	1.024	1.009	1.039
	Sex = 1	0.405	0.557	0.455	1.499	0.518	4.341
	Sex = 2	0^b^	.	.	.	.	.
	Age = 1	0.096	0.026	0.872	1.101	0.341	3.558
	Age = 2	3.92	19.736	<0.001	50.406	8.941	284.172
	Age = 3	0^b^	.	.	.	.	.
	BMI = 1	−1.621	2.912	0.088	0.198	0.031	1.272
	BMI = 2	0^b^	.	.	.	.	.
IV	Intercept	−37.698	27.19	<0.001			
	LDL	−3.609	133.237	<0.001	0.027	0.015	0.05
	N-HDL	2.809	170.689	<0.001	16.592	10.886	25.287
	TG	−0.213	1.202	0.273	0.808	0.552	1.183
	ApoB	2.811	51.734	<0.001	16.630	7.731	35.776
	ApoA1	−0.313	2.78	0.095	0.731	0.506	1.056
	UA	0.005	1.489	0.222	1.006	0.997	1.014
	ApoE	0.299	3.373	0.066	1.349	0.98	1.857
	APTT	0.289	6.103	0.013	1.335	1.061	1.678
	PT	0.45	3.412	0.065	1.569	0.973	2.53
	D-D	1.171	37.783	<0.001	3.227	2.221	4.688
	PLT	0.011	1.805	0.179	1.011	0.995	1.029
	Sex = 1	0.548	0.75	0.387	1.729	0.501	5.975
	Sex = 2	0	.	.	.	.	.
	Age = 1	−0.355	0.201	0.654	0.701	0.149	3.308
	Age = 2	6.348	42.517	<0.001	571.533	84.785	3852.674
	Age = 3	0^b^	.	.	.	.	.
	BMI = 0	−3.024	8.041	0.005	0.049	0.006	0.393
	BMI = 1	0^b^	.	.	.	.	.

“b” means that the item is a reference item and is therefore set to 0

## Discussion

At present, NONFH is still recognized as a refractory disease in the world, with young and middle-aged people in the majority, and about 80% of them involve bilateral hip joints [9], and most of the patients suffer from recurrent pain and severe dysfunction for a long period, even losing their mobility, and eventually have to undergo total hip arthroplasty.In the younger age group, it is important to identify risk factors for morbidity and cautiously pursue hip-conserving surgical treatments to alleviate symptoms [13]. At present, ARCO staging changes and imaging changes of femoral head collapse are mainly used to evaluate the progression of femoral head necrosis [14], and several studies have reported that the risk factors influencing the occurrence of NONFH endpoints include: The location, extent, and morphology of necrotic areas of the femoral head, the signal characteristics of necrotic areas, and the strength of the femoral neck [15, 16], and there are no articles on whether there is a relationship between clinical laboratory indicators, such as coagulation indicators, lipid metabolism indicators, and bone metabolism indicators, and the progression of NONFH. There are no articles to indicate whether there is a relationship between clinical laboratory indicators, such as coagulation indicators, lipid metabolism indicators, and bone metabolism indicators, and the course of NONFH, and this study focuses on the above indicators to explore the risk factors for the development of NONFH other than imaging factors.

The results of this study showed that elevated LDL was negatively correlated with NONFH disease progression, and in the alcohol group, LDL levels were higher in ARCO stage IV than in ARCO stage II (*P* < 0.05);N-HDL and ApoB in the alcohol group, hormone group, and idiopathic group increased significantly with disease progression (*P* < 0.001), and the results of multivariate logistic regression analysis showed that for every 1-unit increase in N-HDL, while other indicators remained unchanged, the probability of ARCO stage III and IV was 5-fold and 16. 592-fold higher than that of stage II, and that of ApoB was 5.009-fold and 16.63-fold higher, respectively, indicating that N-HDL and ApoB are risk factors for disease progression in NONFH.Currently, there are more views that hyperlipidemia is a risk factor for the induction of femoral head necrosis in people who abuse hormones and long-term alcoholism, but in the present study, although it was observed that the levels of LDL and TG in the alcohol and hormone groups were elevated higher than normal with the progression of the disease, there was no positive correlation with the actual disease progression (*P* > 0.05),In general, elevated serum LDL will be deposited in the arterial walls of blood vessels, gradually forming atherosclerotic plaques and affecting the flow rate of blood to the femoral head, but the factors affecting elevated serum LDL and TG are complex, It has been suggested that the congenital presence of hyperlipidemia may not increase the risk of osteonecrosis in rabbits and that a diet rich in lanolin may also be a protective factor [17].Another cause of abnormally elevated lipid metabolism markers may be the patient’s comorbidities with varying degrees of liver dysfunction or fatty liver. It has been previously reported [18, 19] that liver dysfunction or definite alcoholic/non-alcoholic fatty liver was observed in patients with alcohol-induced NONFH and steroid-induced NONFH, suggesting that any hepatic impairment (including fatty liver as well as oxidative stress, etc.) is a risk factor for NONFH. ) is a risk factor for NONFH, and this was supported by the fact that the abnormal elevation of lipid metabolism indices such as LDL and TG was observed to be higher in the alcohol and hormone groups than in the idiopathic group. This is also supported by the fact that the alcohol and hormone groups had higher LDL and TG levels than the idiopathic group.In addition, the present study observed the changes of N-HDL in different etiologic groups in different ARCO stages, which is consistent with the results of a cross-sectional study [4]that N-HDL is a reliable indicator in lipid metabolism metrics for assessing the progression of NONFH, N-HDL is also a major risk factor for atherosclerotic disease and supports the increased risk of secondary coronary heart disease in NONFH patients.In conclusion, the relationship between changes in various indices of lipid metabolism and the progression of NONFH remains controversial, and further studies of dyslipidemia and obesity and comorbidities between different etiologies are needed.

In terms of bone metabolism, this study found that alkaline phosphatase generally tended to increase with disease progression in the different etiologic groups, but the difference between the groups was not statistically significant (*P* > 0.05);In contrast, elevated bone-specific alkaline phosphatase, one of the phenotypic markers of osteoblasts, is generally seen in metabolic bone diseases with high turnove [20].A bone histopathologic study of a rabbit model of SONFH [21] found that serum b-ALP levels began to increase from the 4th week of modeling and decreased by the 8th week, while the number of osteoblasts decreased significantly from the 4th week.In the present study, it was observed that b-ALP levels in the alcohol and hormone groups were significantly higher in ARCO stage III than in the other two stages, and serum b-ALP levels were lowest in stage IV (*P* < 0.05), and there was also a trend in the idiopathic group, but the difference between the different stages was not significant (*P* = 0.545). Decreased NBAP implies increased apoptosis of periarticular osteoblasts, and prolonged exposure to such an inflammatory environment severely affects hip angiogenesis as well as bone repair and regeneration [22]. Taken together with the results of the present study, this suggests that with the progression of NONFH, the destruction of the patient’s local bone tissues, as well as the continuous erosion of inflammatory factors, there will be a transient enhancement of bone remodeling in stage III, followed by a further increase in bone resorption.

The present study again validated PLT as a risk factor for disease progression in NONFH. Multiple Logistic regression analysis showed that for every 1-unit decrease in PLT, the odds of ARCO stage progression to stages III and IV were 1.024 and 1.011 times higher, respectively.In different groups, PLT levels were elevated in ARCO stage III compared to ARCO stage II, whereas PLT was significantly lower in ARCO stage IV than in stage II (*P* < 0.05).The main functions of platelets include coagulation and repair damaged blood vessels [23], analyzed with the results, this situation may be because when NONFH progresses, the blood supply to the femoral head is damaged, and at this time, platelets are stimulated to start the vascular repair, The surface viscosity increases and coagulates into a cluster, and at the same time participates in coagulation, forming clots with the blood cells, so it may be possible to have a transient increase in ARCO III stage and then decrease in the Therefore, there may be a transient increase in ARCO III and then decrease in ARCO III.Platelet-rich plasma (PRP) has been widely used in recent years in the biologic treatment of musculoskeletal disorders with the ability to promote injury repair and healing [24], and the application of PRP arthroplasty injections, alone or in combination, to the treatment of NONFH is currently at the stage of exploratory trials.In addition to PLT, this study also found that APTT was positively correlated with NONFH disease progression, and among the different groups, the reduction in APTT was smaller in ARCO stage III than in stage II (*P* < 0.05),The APTT is commonly used as a clinical screening test for the coagulation activity of the endogenous coagulation system, and a shortening usually indicates increased coagulation factor VIII activity as well as a hypercoagulable state of the blood, etc. The results indicate that this stage is weaker than stage II hypercoagulability, and there is a rich perfusion of blood flow; Previous studies have shown that thrombus is a risk factor for NONFH and that perfusion in the necrotic area of the femoral head significantly influences the progression of osteonecrosis, with ARCO stage III being faster than stage II [25]. PT also showed that ARCO stage IV was shorter than stages II and III and stage II was shorter than stage III in different groups, indicating that stage IV was more prone to hypercoagulable and thrombotic states compared to the first two stages.Another indicator of hypercoagulability in the body, D-dimer, was found to be 3.227 times more likely to progress to ARCO stage IV than stage II for every 1-unit increase in D-dimer in this study, and the results once again demonstrated that the progression of femoral head necrosis is closely related to the hypercoagulable state of the blood. A study using matrix-assisted laser-resolved ionization time-of-flight mass spectrometry and gene sequencing to analyze 146 patients with femoral head necrosis [26] showed that the rs6020 G-to-A polymorphism was detected in 88.9% of the patients, and 87.6% of the patients had an abnormal hypercoagulable state, Cause analysis revealed that when patients with the rs6020 polymorphism were exposed to risk factors such as alcohol and hormone abuse, it would leads to abnormal hypercoagulation, which in turn would lead to thromboembolism of the femoral head. This shows that changes in some coagulation function indices are closely related to the progression of NONFH.

There are some limitations in this study, first of all, the study retrospectively analyzed the medical records of patients who visited the hospital in the past 4 years, and most of the patients came to the clinic mainly because of obvious hip symptoms, and according to ARCO staging, most of them were in stage III or above, which resulted in unequal number of cases in each staging; Second, with exposure factors such as hormones and alcohol, The patients were mostly comorbid with other underlying diseases or had varying degrees of liver injury, which may have affected the results of this study, and this study did not stratify whether the liver injury present in each group was alcoholic or hormonal.In future studies, we plan to combine imaging data from patients with different exposure factors for further analysis, as well as to increase the sample size and control for the influence of underlying disease on the findings, in order to further analyze the risk factors for progression of NONFH disease.

## Conclusion

In the present study, it was observed that N-HDL, ApoB, APTT, and PLT in lipid metabolism and coagulation indices in different etiologic groups were risk factors for disease progression of NONFH, whereas LDL might be a protective factor for NONFH progression.NONFH patients with abnormal blood hypercoagulability and dyslipidemia should be taken seriously in clinical practice and disease progression should be considered. The bone turnover index b-ALP was specific in different ARCO stages in the alcohol and hormone groups, but the correlation with disease progression in NONFH needs further study.

### Electronic supplementary material

Below is the link to the electronic supplementary material.

Supplementary Material 1

## Data Availability

Data is provided within the manuscript or supplementary information files.
